# Conversion of Multi-layered MoTe_2_ Transistor Between P-Type and N-Type and Their Use in Inverter

**DOI:** 10.1186/s11671-018-2721-0

**Published:** 2018-09-21

**Authors:** Junku Liu, Yangyang Wang, Xiaoyang Xiao, Kenan Zhang, Nan Guo, Yi Jia, Shuyun Zhou, Yang Wu, Qunqing Li, Lin Xiao

**Affiliations:** 1 Nanophotonics and Optoelectronics Research Center, Qian Xuesen Laboratory of Space Technology, China Academy of Space Technology, Beijing, 100094 China; 2 State Key Laboratory of Low-Dimensional Quantum Physics, Department of Physics and Tsinghua-Foxconn Nanotechnology Research Center, Tsinghua University, Beijing, 100084 China; 3 Department of Physics, Tsinghua University, Beijing, 100084 China

**Keywords:** MoTe_2_, P-type, N-type, Absorbates, Vacancies

## Abstract

Both p-type and n-type MoTe_2_ transistors are needed to fabricate complementary electronic and optoelectronic devices. In this study, we fabricate air-stable p-type multi-layered MoTe_2_ transistors using Au as electrode and achieve the conversion of p-type transistor to n-type by annealing it in vacuum. Temperature-dependent in situ measurements assisted by the results given by first-principle simulations indicate that n-type conductance is an intrinsic property, which is attributed to tellurium vacancies in MoTe_2_, while the device in air experiences a charge transfer which is caused by oxygen/water redox couple and is converted to air-stable p-type transistor. Based on p-type and n-type multi-layered MoTe_2_ transistors, we demonstrate a complementary inverter with gain values as high as 9 at *V*_DD_ = 5 V.

**Electronic supplementary material:**

The online version of this article (10.1186/s11671-018-2721-0) contains supplementary material, which is available to authorized users.

## Background

Graphene and similar two-dimensional (2D) materials exist in bulk form as stacks of strongly bonded layers with weak interlayer attraction, allowing itself to be exfoliated into atomically thin layers, which have opened up new possibilities for the exploration of 2D physics as well as that of new material applications [1–9]. Of them, semiconductor transition metal dichalcogenides (TMDs) exhibit sizeable bandgaps [2, 3, 10, 11]. In addition, these 2D TMD flakes are flexible and free of dangling bonds between adjacent layers [12, 13]. These unique properties make TMDs promising candidates to construct electronic and optoelectronic devices [2–4, 14], such as next-generation field-effect transistor (FET) at sub 10 nm [15], inverter [16–22], and on-chip light-emitting diode (LED) [23–25] and Van der Waals heterostructure devices [4, 5, 26–28].

2H-type molybdenum ditelluride (2H-MoTe_2_) is one of the typical 2D TMDs, which has an indirect bandgap of 0.83 eV in bulk form [29] and a direct bandgap of 1.1 eV when it is thinned to a monolayer [30]. 2H-MoTe_2_ has been explored for applications in spintronics [31], FET [32–34], photodetector [35–38], and solar cell [39]. Like most 2D materials, multi-layered 2H-MoTe_2_ has very high surface-to-volume ratio, making it sensitive to various influences in the surrounding environment. Thus, it is difficult to obtain its intrinsic properties. The surface and interface of 2D materials and related devices have always been research hotspots in order to achieve higher performance. Here, we fabricate a multi-layered 2H-MoTe_2_ transistor, whose source and drain electrode layers are fabricated, and then, a multi-layered MoTe_2_ sample is transferred to bridge the source and drain electrodes as a transistor channel. The whole MoTe_2_ sample is exposed in air, including the channel and contact part, which is advantageous to investigating the influence of absorbates on charge transport properties of multi-layered MoTe_2_ transistor. Measurements of vacuum- and temperature-dependent charge transport are conducted. The experimental data show that multi-layered MoTe_2_ transistor is an n-type in terms of intrinsic conductance. However, the device exposed in air can be doped by absorbates and converted to air-stable p-type transistor. We infer that the intrinsic n-type conductance of multi-layered MoTe_2_ transistor is attributed to tellurium (Te) vacancies, which is confirmed by density functional theory (DFT) calculations. The conversion to p-type conductance in air can be explained by the fact that oxygen and water absorbed in air can induce electron transfer from MoTe_2_ to oxygen/water redox couple, which converts n-type multi-layered MoTe_2_ transistor to p-type. Finally, based on the n-type and p-type multi-layered MoTe_2_ transistors, we demonstrate a complementary inverter, which shows symmetric input/output behavior and gain values of 9 at *V*_DD_ = 5 V.

## Results and Discussion

**Fig. 1:**
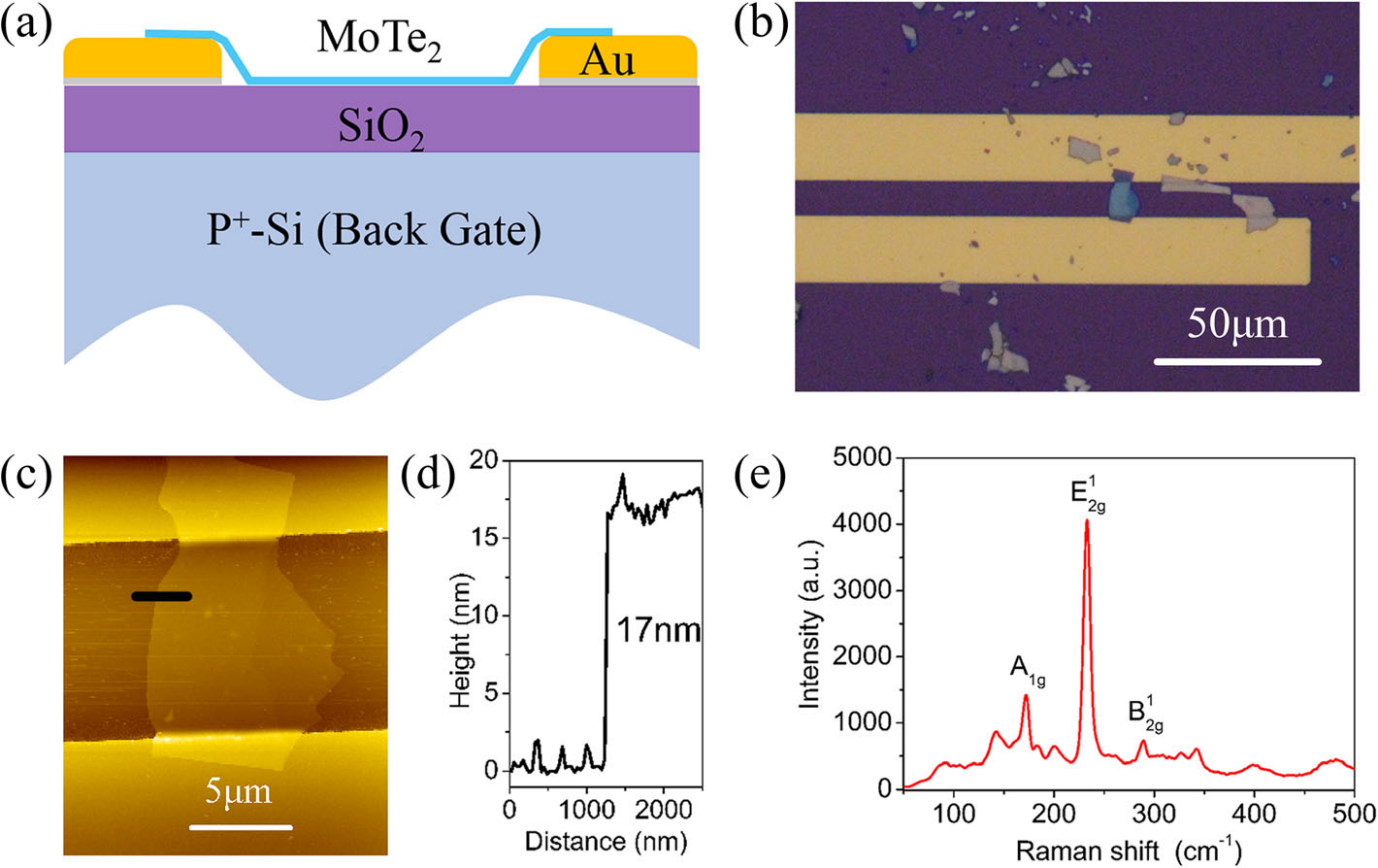
Multi-layered MoTe_2_ transistor and its properties. **a** Illustration of MoTe_2_ transistor diagram. **b** Optical image of one of the fabricated transistors composed of multi-layered MoTe_2_ channel and SD Cr/Au electrodes. **c** AFM image of the transistor channel in **b**. **d** Height profile of the multi-layered MoTe_2_. **e** Raman spectrum of the multi-layered MoTe_2_ in the transistor channel

Different from the previously reported multi-layered MoTe_2_ transistor, our device diagram is shown in Fig. 1a. We first fabricate source-drain (SD) electrodes composed of Cr/Au film on SiO_2_/p^+^-Si substrate. Then, one of the multi-layered MoTe_2_ samples prepared on another SiO_2_/ p^+^-Si substrate is transferred to bridge the source-drain electrodes as transistor channel. The MoTe_2_ sample made by this method is clean and free of polymer contamination in device fabrication. In addition, the whole MoTe_2_ sample is exposed in air, including the channel and contact part, making it more convenient to remove absorbates and obtain intrinsic conductance of multi-layered MoTe_2_ transistor. An optical image of a fabricated multi-layered MoTe_2_ transistor is shown in Fig. 1b, with a channel length of 10 μm. The MoTe_2_ channel is characterized by atomic force microscopy (AFM) (see Fig. 1c). Height profile (see Fig. 1d) obtained from the mark in AFM image indicates that the thickness of MoTe_2_ sample is about 17 nm (composed of 24 monolayer MoTe_2_) [40]. The characteristic Raman-active modes of A_1g_ (172 cm^−1^), E^1^_2g_ (233 cm^−1^), and B^1^_2g_ (289 cm^−1^) are clearly observed as shown in Fig. 1e, indicating the good quality of 2H-MoTe_2_ after the transfer process [41].

**Fig. 2:**
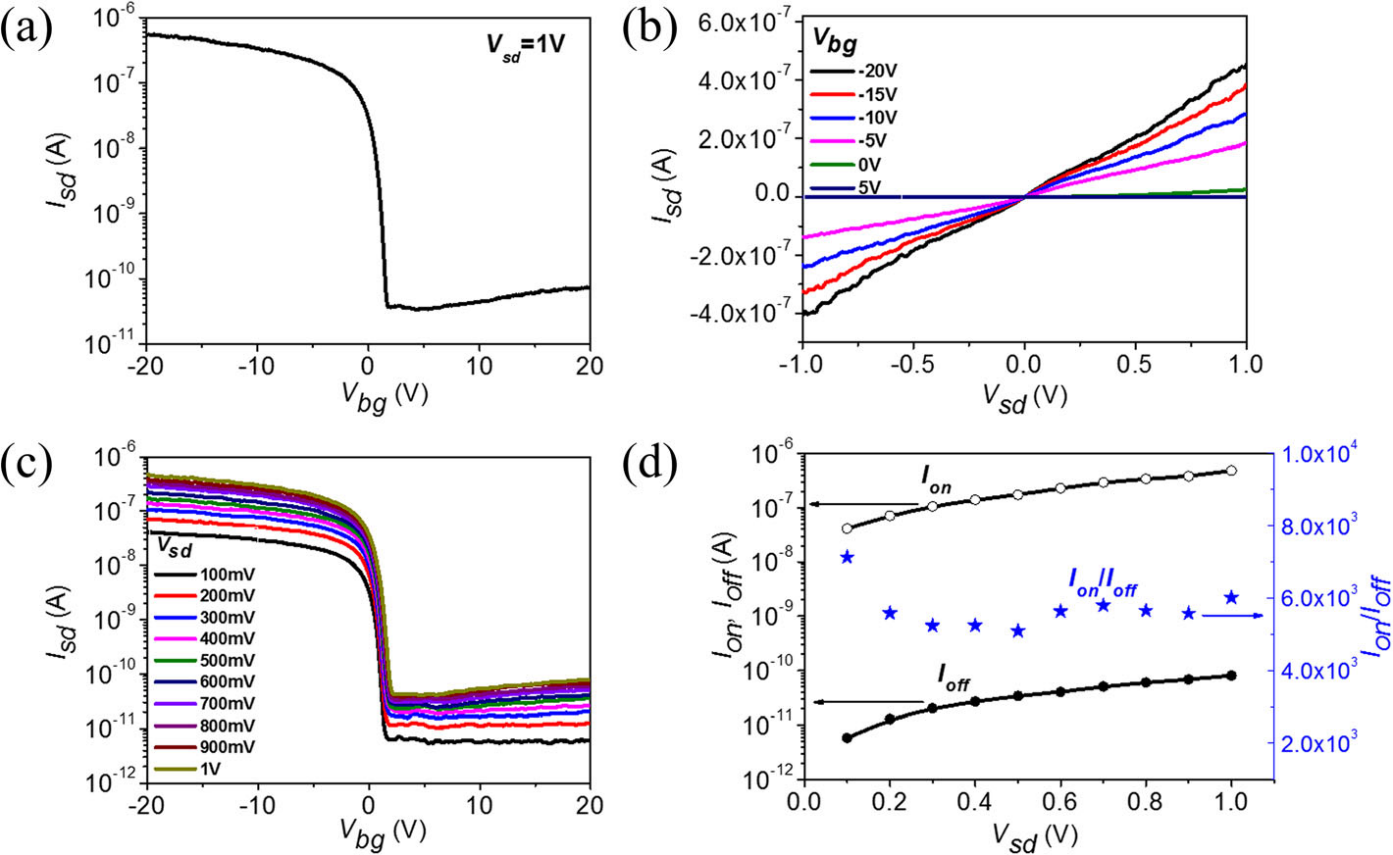
Electric properties of multi-layered MoTe_2_ transistor in air at RT. **a** Transfer characteristics of MoTe_2_ transistor at *V*_sd_ = 1 V in air. **b** Output characteristics of MoTe_2_ transistor at *V*_bg_ = − 20 V, − 15 V, − 10 V, − 5 V, 0 V, and 5 V. **c** Transfer characteristics of MoTe_2_ transistor at different *V*_sd_. **d** On-current, off-current, and on-off current ratio as function of *V*_sd_

The fabricated back-gated multi-layered MoTe_2_ transistors are measured using Agilent B1500A semiconductor analyzer in Lakeshore probe station, which can be pumped to a base pressure of 1 × 10^−5^ mbar and realize 9~350 K temperature adjustment. Figure 2 shows the electric properties of a multi-layered MoTe_2_ transistor in air at room temperature (RT). The transfer characteristics at source-drain voltage *V*_sd_ = 1 V in Fig. 2a show that the transistor is in on-state at negative gating voltage and in off-state at positive gating voltage. The transform voltage from on-state to off-state is nearly zero, which is a typical p-type transistor characteristic. Replicate measurements show the same electric gating characteristics (see Additional file 1: Figure S1). Four other multi-layered MoTe_2_ transistors also demonstrate similar p-type electric gating characteristics as shown in Additional file 1: Figure S2. We also prepare other devices with thicknesses of 5 nm, 38 nm, and 85 nm as shown in Additional file 1: Figure S3. When the MoTe_2_ thicknesses are 5 nm and 38 nm, both the prepared devices show p-type conductance but with small on-current compared with the device in Fig. 2 and Additional file 1: Figure S2. As the thickness increases to 85 nm, the gating effect disappears as shown in Additional file 1: Figure S3 (l). These data show that p-type conductance is universal in air for multi-layered MoTe_2_ transistor. From transfer characteristics in Fig. 2a, we can obtain the on-off ratio, subthreshold swing (SS), and field-effect mobility (μ), which are 6 × 10^3^, 350 mV/dec, and 8 cm^2^/V·s, respectively.

Figure 2b shows the output characteristics of multi-layered MoTe_2_ transistor at back-gate voltage *V*_bg_ = − 20 V, − 15 V, − 10 V, − 5 V, 0 V, and 5 V. As seen, the response is essentially linear, especially at low biased voltage of *V*_sd_, which indicates there is negligible effective Schottky barrier height (*Φ*_SB_) between Au and MoTe_2_ in air. The transfer characteristics at different source-drain biased voltages as shown in Fig. 2c indicate that the on-current increases linearly with biased voltage *V*_sd_, shown in Fig. 2d, which coincides with the output characteristics. Meanwhile, the off-current increases and on-off ratio decreases as *V*_sd_ increases. This can be attributed to trap state in MoTe_2_ channel from absorbates and interface state. The hysteresis in transfer characteristics (see Additional file 1: Figure S4) further confirms the existence of trap state in MoTe_2_ transistor [42–45].

**Fig. 3:**
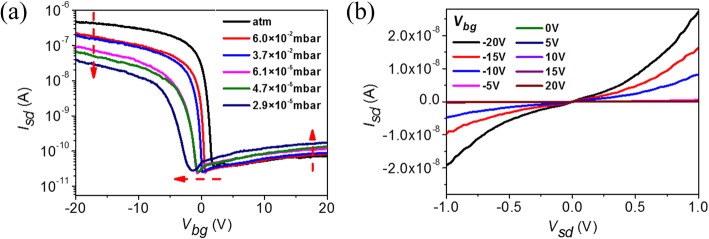
P-type electric properties of multi-layered MoTe_2_ transistor in vacuum. **a** RT transfer characteristics of a p-type MoTe_2_ transistor at *V*_sd_ = 1 V as a function of vacuum. **b** RT output characteristics of a p-type MoTe_2_ transistor at different *V*_bg_ in 2.9 × 10^−5^ mbar vacuum

We further investigate the p-type conductance of multi-layered MoTe_2_ transistor at different vacuums. This is helpful to understand the influence of absorbed oxygen and water on the charge transport properties. Figure 3a shows the transfer characteristics at *V*_sd_ = 1 V as a function of vacuum (“atm” corresponds to atmosphere). The major changing tendencies are clearly indicated by red arrows, which is similar to that shown in carbon nanotube transistor [44]. First, the on-current decreases as vacuum increases, which is partially due to the shift of threshold voltage caused by absorbates but mainly due to device resistance increase as absorbates decrease, including channel and contact resistance. The nonlinear output characteristics as shown in Fig. 3b indicate the enhanced effective Schottky barrier between Au and MoTe_2_ in 2.9 × 10^−5^ mbar vacuum, which suggests that the effective Schottky barrier height is modified by absorbates in air. Second, the off-current at positive voltage gating increases with the vacuum, which means that electron conductance increases as absorbates decrease and suggests that n-type conductance is suppressed in multi-layered MoTe_2_ transistor by absorbates in air.

**Fig. 4:**
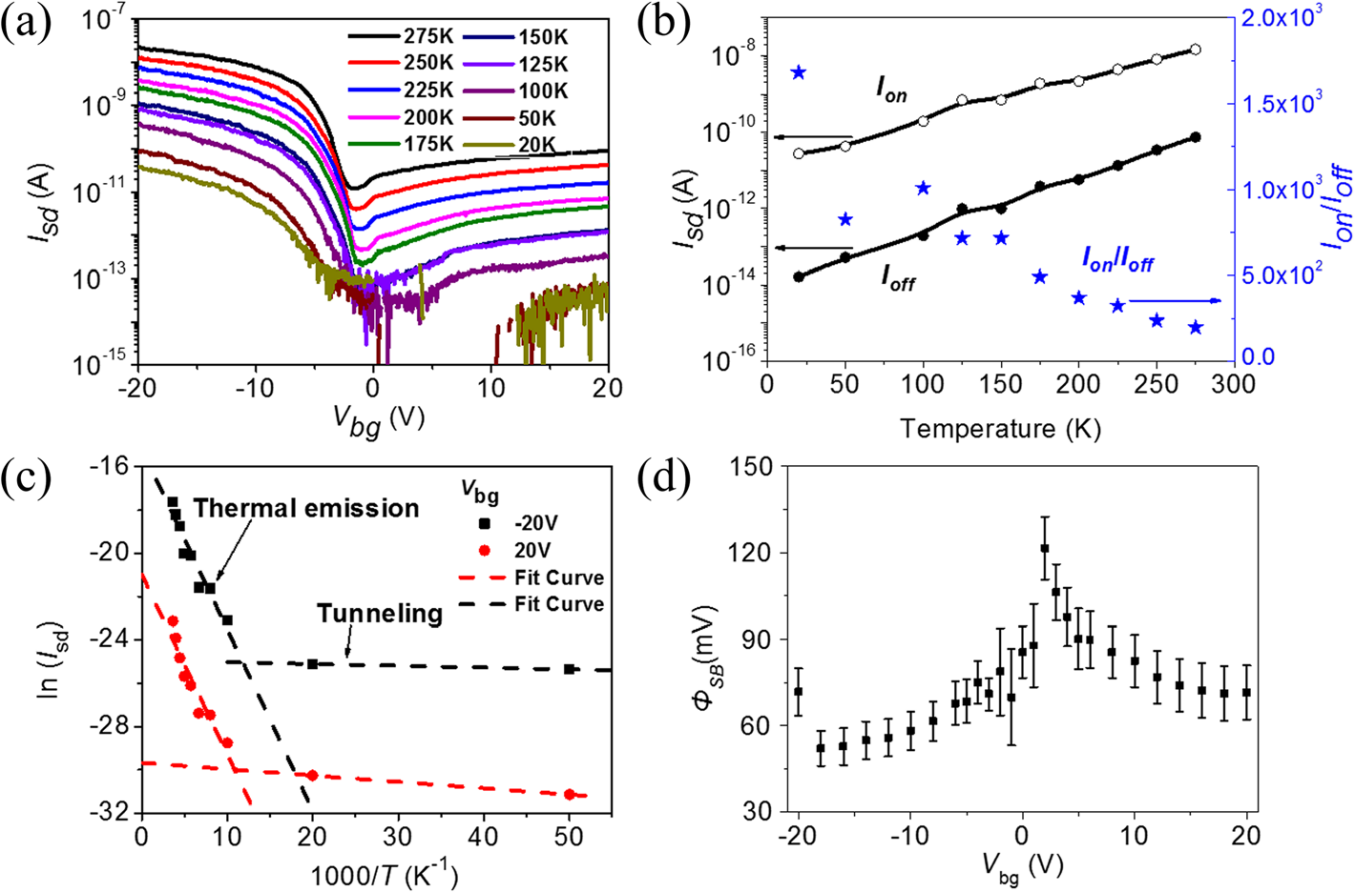
Temperature-dependent electric properties of a p-type multi-layered MoTe_2_ transistor. **a** Transfer characteristics of MoTe_2_ transistor at *V*_sd_ = 1 V as a function of temperature. **b** On-current, off-current, and on-off current ratio as a function of temperature. **c** Arrhenius plot of the source-drain current as a function of temperature at *V*_sd_ = 1 V and *V*_bg_ = − 20 V and 20 V, respectively. **d** Maps of effective Schottky barrier heights *Φ*_SB_ as a function of back-gate voltage

Although the on-current decreases and off-current increases after eliminating partial absorbates in vacuum, the multi-layered MoTe_2_ transistor still exhibits p-type conductance. Furthermore, p-type conductance maintains at low temperature as shown in Fig. 4a. This temperature-dependent electric property helps us to further elucidate the charge transport mechanism and extract the effective Schottky barrier height of p-type MoTe_2_ transistor. Figure 4a gives the transfer characteristics at biased voltage *V*_sd_ = 1 V as temperature varies from 20 to 275 K. Both on-current and off-current decrease as temperature decreases, and the on-off ratio increases at low temperatures as shown in Fig. 4b. Arrhenius plot of the source-drain current *I*_sd_ at back-gate voltage *V*_sd_ = − 20 V and 20 V in Fig. 4c indicates the thermal emission and tunneling contribution for charge transport [46]. When temperature is higher than 100 K, a clear thermal emission region is observed in both negative and positive gating voltages, and the tunneling current dominates when temperature is below 100 K. That is why both on-current and off-current decrease as temperature decreases. Based on the thermal emission current observation and the relationship of ${I}_{\mathrm{sd}}\sim {e}^{-{q\varPhi}_{SB}/ kT\operatorname{}}$, where *k* is the Boltzmann constant and *T* is temperature, we extract the effective Schottky barrier height *Φ*_SB_ as a function of gate voltage at *V*_sd_ = 1 V, as shown in Fig. 4d. The effective Schottky barrier heights *Φ*_SB_ in both on- and off-state are smaller than 120 mV.

**Fig. 5:**
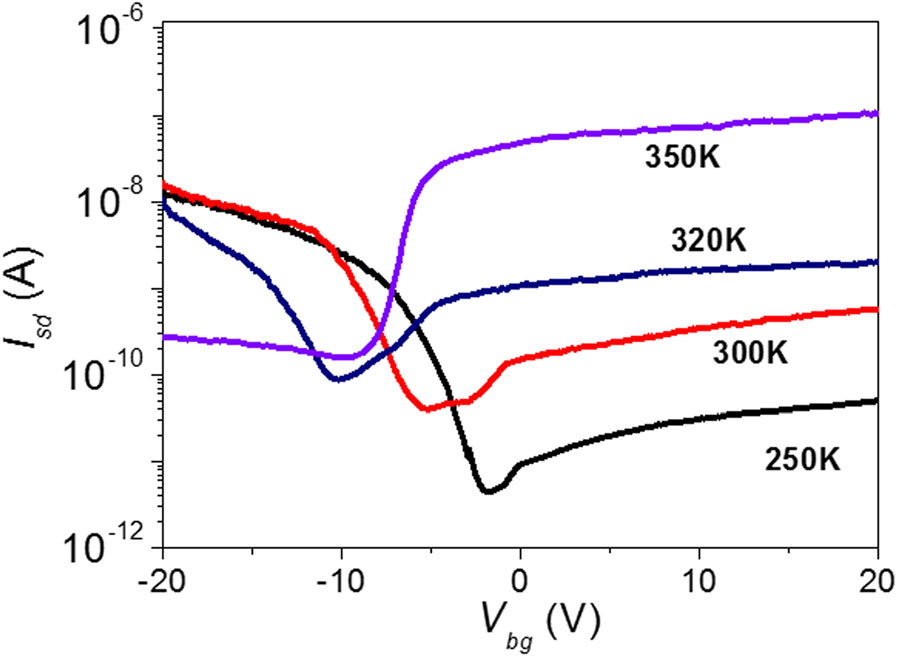
The transfer characteristics of multi-layered MoTe_2_ transistor as a function of temperature in vacuum

Vacuum and low temperature make it difficult to desorb the absorbates completely. The residual absorbates still work and alter the conductance of multi-layered MoTe_2_ transistor. In order to further desorb the absorbates on MoTe_2_ transistor, we heat the device to 350 K in vacuum and carry out in situ electric property measurements. Figure 5a shows the transfer characteristics of MoTe_2_ transistor as it is heated from 250 to 350 K. As seen, the electron conductance at positive gate voltage is enhanced, while hole conductance at negative gate voltage is reduced as temperature increases. At temperature *T* = 250 K, the device shows a typical p-type conductance. But when temperature increases to *T* = 350 K, the device is converted to n-type, which is in off-state at negative gate voltage and in on-state at positive gate voltage. Its on-off ratio, subthreshold swing (SS), and field-effect mobility (μ) are 3.8 × 10^2^, 1.1 V/dec, and 2 cm^2^/V·s, respectively.

**Fig. 6:**
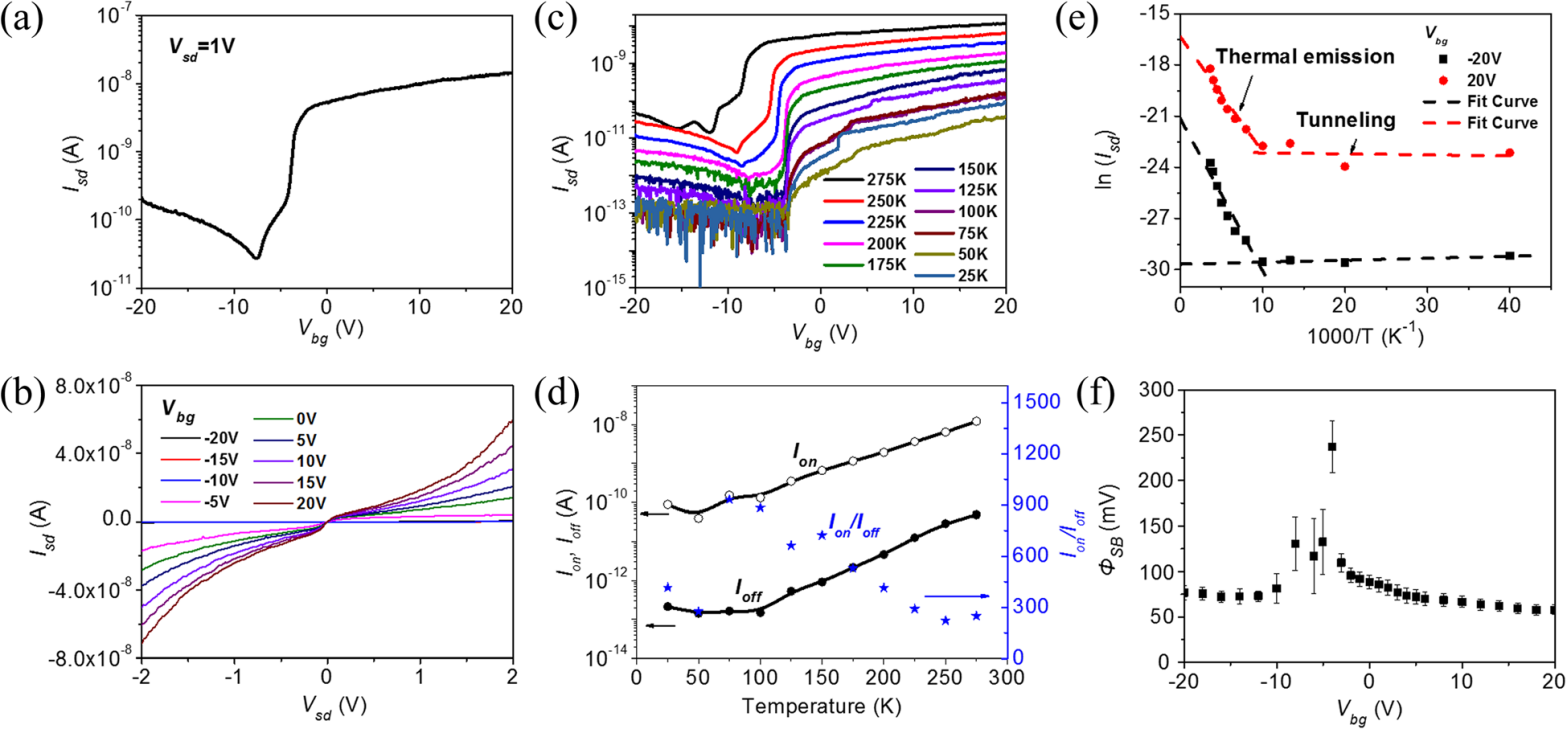
N-type multi-layered MoTe_2_ transistor properties in vacuum. **a** RT transfer characteristics of MoTe_2_ transistor at *V*_sd_ = 1 V. **b** RT output characteristics of MoTe_2_ transistor at different back-gate voltage. **c** Transfer characteristics of MoTe_2_ transistor as a function of temperature. **d** On-current, off-current, and on-off current ratio of MoTe_2_ transistor as a function of temperature. **e** Arrhenius plot of the *I*_sd_ at *V*_sd_ = 1 V and *V*_bg_ = − 20 V and 20 V, respectively. **f** Maps of effective Schottky barrier heights *Φ*_SB_ as a function of *V*_bg_

The n-type conductance of a MoTe_2_ transistor is stable in vacuum. The device is kept in probe station in 2 × 10^−5^ mbar vacuum at RT for 12 h after heating. Then, the measurements of electric properties are conducted. As shown in Fig. 6a, the transfer characteristics are still in off-state at negative gate voltage and in on-state at positive gate voltage, demonstrating typical n-type transistor properties. Similar transformations are realized in the other two samples as shown in Additional file 1: Figure S5 (a) and (b). Furthermore, we anneal two samples at 523 K using a high-temperature chemical vapor deposition system for 2 h in Ar gas at 3 mbar vacuum. They both change from p-type to n-type as shown in Additional file 1: Figure S5 (c) and (d). Figure 6b shows the output characteristics of an n-type MoTe_2_ transistor at different back-gate voltages, which is clearly nonlinear, especially at low biased voltage *V*_sd_, different from that in Fig. 3b, indicating the existence of enhanced effective Schottky barrier height between MoTe_2_ and Au electrode after being heated to remove absorbates. Figure 6c shows the temperature-dependent transfer characteristics of n-type multi-layered MoTe_2_ transistor. As seen, when temperature decreases from 275 to 25 K, the on-current and off-current both decrease as shown in Fig. 6c, d. Arrhenius plot of the source-drain current *I*_sd_ in Fig. 6e shows that thermal emission and tunneling current are still the main charge transport mechanism in n-type multi-layered MoTe_2_ transistor. The effective Schottky barrier height thus obtained is smaller than 250 meV. Considering the work function of Au (5.2 eV) and MoTe_2_ (4.1 eV), the effective Schottky barrier height for electron is as high as 1.1 eV in ideal condition. The difference may be from the Fermi level pinning effect in 2D materials [47].

**Fig. 7:**
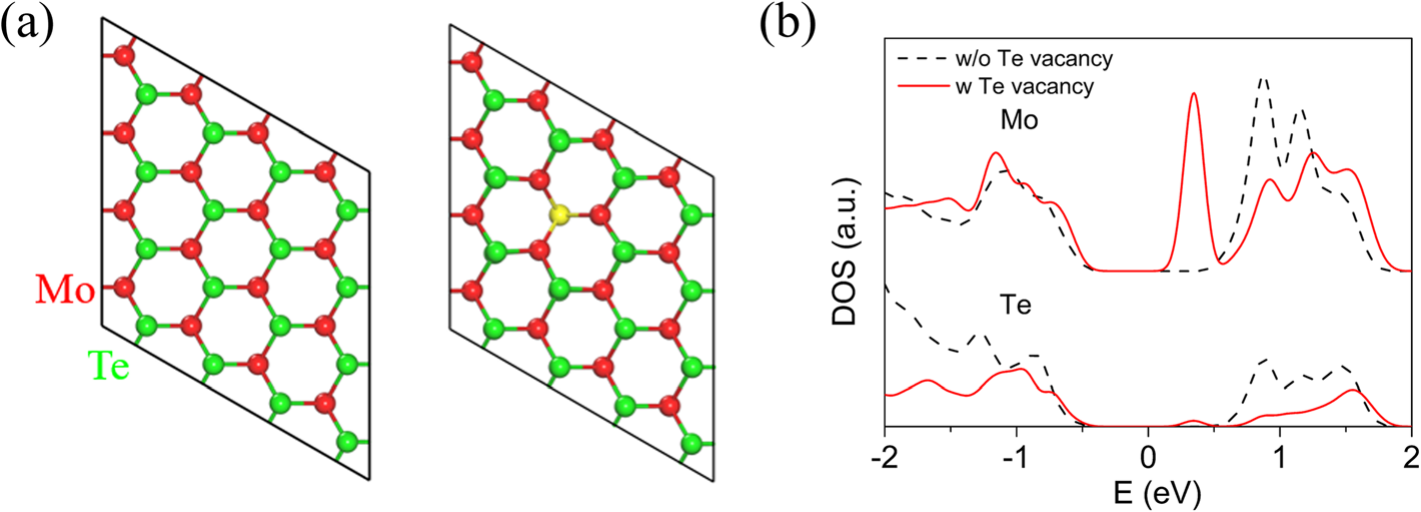
Te vacancy in MoTe_2_. **a** 4 × 4 ML MoTe_2_ supercells in an ideal phase and with a Te vacancy. The vacancy site is marked in yellow. **b** Partial density of states (PDOS) of Mo site adjacent to Te vacancy and nearest Te site to a Te vacancy in ML MoTe_2_ (red solid), compared to the PDOS in an ideal ML (black dashed)

We also find that the n-type multi-layered MoTe_2_ transistor returns to p-type when it is exposed to air (see Additional file 1: Figure S6). Based on the above experiment data, we infer that n-type conductance is an intrinsic property for multi-layered MoTe_2_ transistor. N-type conductance can be attributed to Te vacancy in MoTe_2_ channel. It is confirmed by DFT calculation as shown in Fig. 7. Figure 7a shows the illustration of the diagram of Te vacancy in monolayer (ML) MoTe_2_, and Fig. 7b shows the corresponding density of states (DOS). Compared with the DOS of MoTe_2_ with perfect crystal structure, Te vacancy induces a defect state near the conduct band edge. Therefore, MoTe_2_ transistor with Te vacancy demonstrates n-type conductance.

**Fig. 8:**
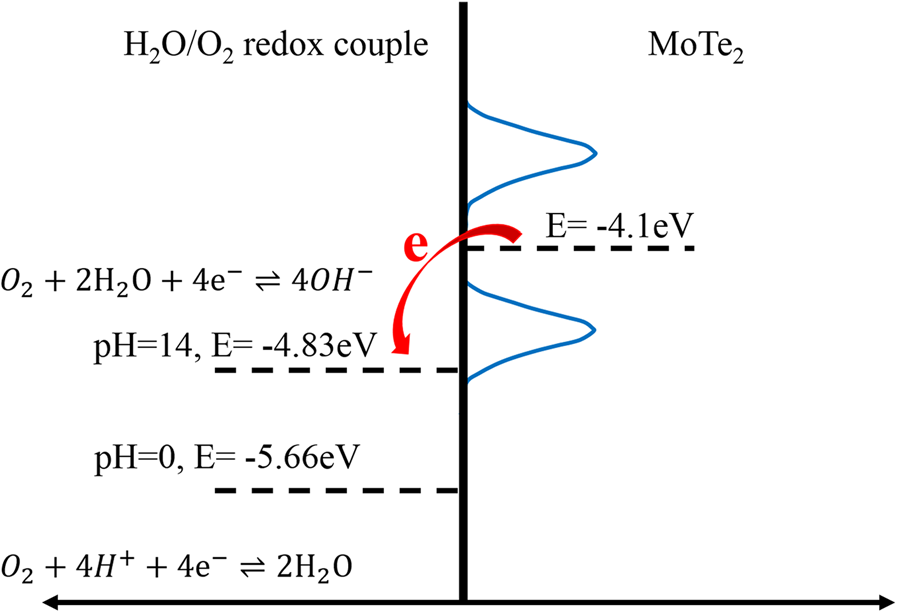
Energy diagram of the water/oxygen redox couple (left) and MoTe_2_ (right); the red arrow indicates the electron transfer direction

When the device is exposed to air, oxygen and water in air are absorbed on the device. It has been verified that the absorbates of oxygen and water can induce p-type doping in organic transistor and graphene-related layer material transistor [44, 48, 49]. It works by oxygen/water redox couple, in which the solved oxygen in water sets the condition for the redox reaction. This process will induce charge transfer between the oxygen/water redox couple and MoTe_2_. Charge transfer direction depends on the work function (or chemical potential) difference. The work function of MoTe_2_ is 4.1 eV, while that of oxygen/water redox couple is larger than 4.83 eV [48]. Figure 8 illustrates the energy diagram of the water/oxygen redox couple and MoTe_2_. Due to the energy level difference, the electrons are injected from MoTe_2_ to oxygen/water redox couple, resulting in hole doping of MoTe_2_ in air.

**Fig. 9:**
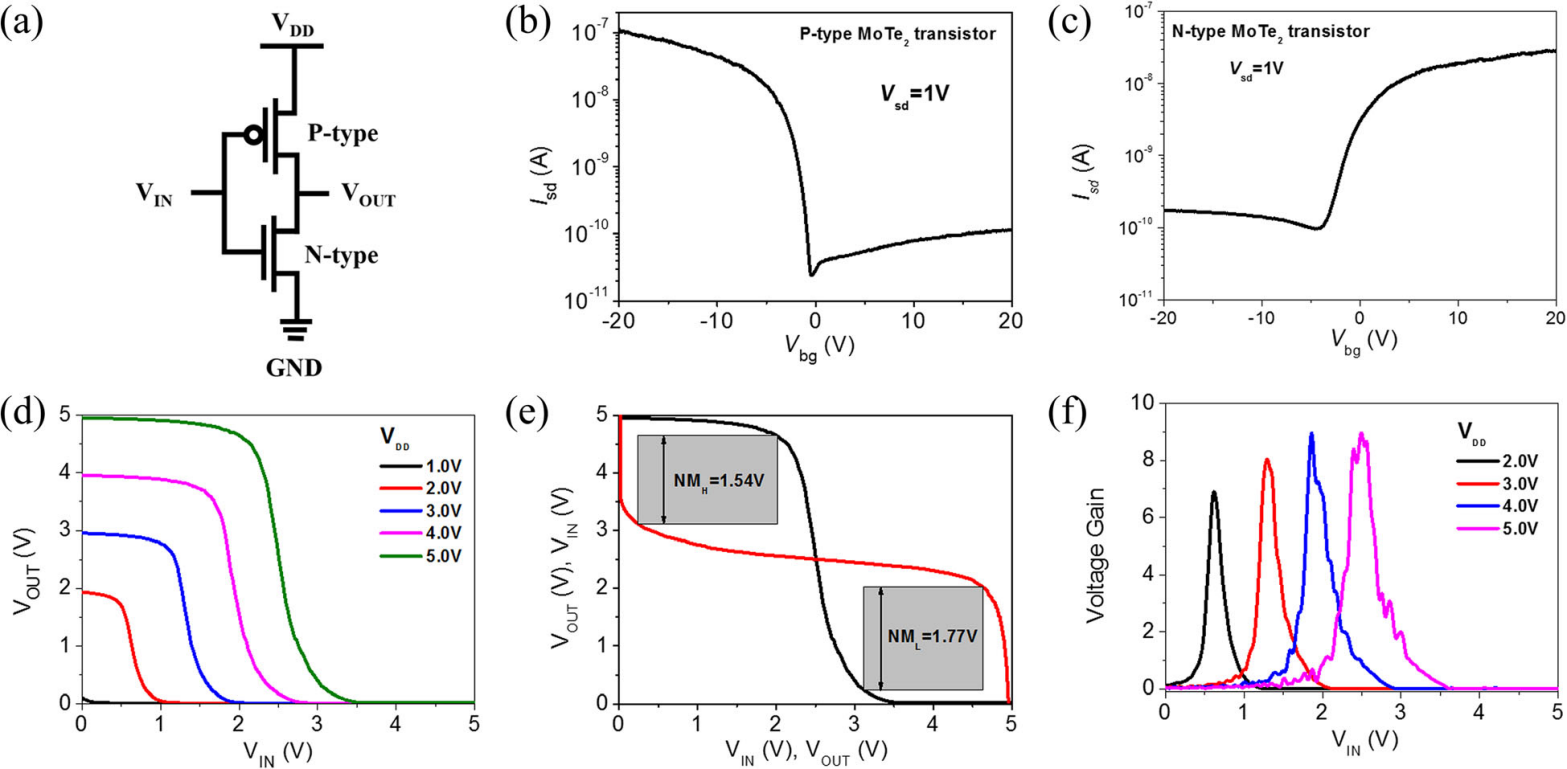
Complementary inverter properties based on p-type and n-type multi-layered MoTe_2_ transistor in 8 × 10^−5^ mbar vacuum. **a** Inverter diagram composed of p-type and n-type MoTe_2_ transistors. Transfer characteristics of p-type (**b**) and n-type (**c**) MoTe_2_ transistor from the inverter. **d** VTC curves of the inverter for *V*_DD_ values varying from 1 to 5 V. **e** VTC curves (black lines) and their mirrors (red lines) at *V*_DD_ = 5 V. **f** *V*_IN_-dependent voltage gains of the inverter at *V*_DD_ = 2 V, 3 V, 4 V, and 5 V

Using the p-type and n-type MoTe_2_ transistors, we explore the construction of a complementary inverter as illustrated in Fig. 9a. A supply voltage of *V*_DD_ is applied to the source (or drain) of p-type transistors, while the source (or drain) of the n-type transistor is grounded. The inverter is measured in 8 × 10^−5^ mbar vacuum in probe station. Figure 9b, c shows the transfer characteristics of p-type and n-type transistors from the inverter, respectively. Figure 9d shows the voltage transfer characteristics (VTC) curves of the inverter when *V*_DD_ varies in the range of 1 to 5 V. The transition voltage is located very near to *V*_DD_/2, which can be attributed to the symmetry between n- and p-type MoTe_2_ transistors. Figure 9e shows the VTC curves (black lines) and their mirrors (red lines) at *V*_DD_ = 5 V. The shaded “eye” area represents the noise margin of the inverter. As seen, the low-level noise margin (NM_L_) and high-level noise margin (NM_H_) are 1.54 V and 1.77 V, respectively, at *V*_DD_ = 5 V. Figure 9f shows *V*_IN_-dependent voltage gains of the inverter at *V*_DD_ = 2 V, 3 V, 4 V, and 5 V which increases with *V*_DD_ and reaches 9 at *V*_DD_ = 5 V.

## Conclusions

In summary, we have fabricated a p-type multi-layered MoTe_2_ transistor by transferring MoTe_2_ onto fabricated source-drain electrode in air. Vacuum- and temperature-dependent in situ charge transport measurements demonstrate that the usual p-type conductance of multi-layered MoTe_2_ transistor is not its intrinsic properties, which is caused by oxygen/water redox couple doping in air. When the MoTe_2_ transistor is heated in vacuum to remove absorbates, it exhibits n-type conductance, which is attributed to tellurium vacancies in MoTe_2_ and is its intrinsic transport property. Both p-type and n-type MoTe_2_ transistors show smaller effective Schottky barrier height, which is partially due to the modification by absorbates. The lowered effective Schottky barrier is beneficial to achieving a high-performance MoTe_2_ transistor. Based on these findings, we fabricate a complementary inverter with gain values as high as 9.

## Methods/Experimental

In order to research the influence of adsorbates on charge transport properties of multi-layered MoTe_2_ transistor, we choose back-gated multi-layered MoTe_2_ transistors and the whole MoTe_2_ sample is exposed to the surroundings. Back-gated multi-layered MoTe_2_ transistors are fabricated as follows. First, source, drain, and gate electrodes are patterned on 300-nm SiO_2_/p^+^-Si substrate using standard UV photolithography techniques, followed by selective etching of 300-nm SiO_2_ beneath the gate electrode and E-beam evaporation of a 5-nm/100-nm Cr/Au film. Second, multi-layered MoTe_2_ samples are prepared on other 300-nm SiO_2_/p^+^-Si by mechanical exfoliation of millimeter-size semiconducting 2H-MoTe_2_ single crystals, which are grown by chemical vapor transport using TeCl_4_ as the transport agent in a temperature gradient of 750 to 700°C for 3 days. Finally, the prepared multi-layered MoTe_2_ samples are transferred onto patterned source-drain electrode using polyvinyl alcohol (PVA) as medium [50]. PVA is dissolved in H_2_O and rinsed with isopropyl alcohol (IPA). Device annealing is carried out in a chemical vapor deposition setup with dry pump. Multi-layered MoTe_2_ samples are identified by an optical microscope, and the corresponding thickness is characterized using SPA-300HV atomic force microscopy (AFM). Raman signals are collected by a LabRAM HR Raman spectrometer with 514-nm wavelength laser excitation in the backscattering configuration using a ×100 objective. The laser power measured from the objective is 2.2 mW. Electrical characterization is performed using a combination of Agilent B1500A semiconductor analyzer with Lakeshore probe station.

The DFT calculations are performed with the projector-augmented wave (PAW) pseudopotential and plane-wave basis set with a cut-off energy of 400 eV implemented in the Vienna ab initio simulation package (VASP) [51]. A vacuum space above 15 Å is chosen in order to eliminate the spurious interaction between periodic images. Enough ***k***-point sampling of 12 × 12 × 1 and 24 × 24 × 1 are used for the structure relaxation and electronic calculations, respectively. The generalized gradient approximation (GGA) with Perew-Burke-Ernzerhof (PBE) functional is adopted [52].

## Additional file

Additional file 1: **Figure S1.** Transfer characteristics of replicate measurements. **Figure S2.** (a)-(d) show the transfer characteristics of other four multi-layered MoTe_2_ transistors in air, respectively. **Figure S3.** MoTe_2_ transistor with different thicknesses. (a)~(d) show the optical image, AFM image, height profile, and transfer characteristics of the device with 5-nm thickness, respectively. (e)~(h) show the optical image, AFM image, height profile, and transfer characteristics of the device with 38-nm thickness, respectively. (i)~(l) show the optical image, AFM image, height profile, and transfer characteristics of the device with 85-nm thickness, respectively. **Figure S4.** Hysteresis behavior of transfer characteristics of multi-layered MoTe_2_ transistor. **Figure S5.** (a)-(d) show the transfer characteristics of four multi-layered MoTe_2_ transistors, respectively. The black line represents the transfer characteristics of as-prepared multi-layered MoTe_2_ transistor in air, and the red line represents that measured from the annealed device. **Figure S6.** (a)-(c) show the transfer characteristics of three multi-layered MoTe_2_ transistors at different conditions, respectively. (DOCX 1011 kb)

## Abbreviation

2D: Two-dimensional
2H-MoTe_2_: 2H-type molybdenum ditelluride
AFM: Atomic force microscopy
DFT: Density functional theory
DOS: Density of states
FET: Field-effect transistor
GGA: Generalized gradient approximation
IPA: Isopropyl alcohol
*I*sd: Source-drain current
LED: Light-emitting diode
NM_H_: High-level noise margin
NM_L_: Low-level noise margin
PAW: Projector-augmented wave
PBE: Perew-Burke-Ernzerhof
PVA: Polyvinyl alcohol
SD: Source-drain
SS: Subthreshold swing
TMDs: Transition metal dichalcogenides
VASP: Vienna ab initio simulation package
*V*_bg_: Back-gate voltage
*V*sd: Source-drain voltage
VTC: Voltage transfer characteristics
*Φ*_SB_: Schottky barrier height
