# Supplementary material for: Conversion of Multi-layered MoTe2 Transistor Between P-Type and N-Type and Their Use in Inverter
Source: Nanoscale Res Lett. 2018 Sep 21;13:291. doi: 10.1186/s11671-018-2721-0 (PMC6150881; doi:10.1186/s11671-018-2721-0)
Supplement: Supplementary file 1 — Figure S1. Transfer characteristics of replicate measurements. Figure S2. (a)-(d) show the transfer characteristics of other four multi-layered MoTe2 transistors in air, respectively. Figure S3. MoTe2 transistor with different thicknesses. (a)~(d) show the optical image, AFM image, height profile, and transfer characteristics of the device with 5-nm thickness, respectively. (e)~(h) show the optical image, AFM image, height profile, and transfer characteristics of the device with 38-nm thickness, respectively. (i)~(l) show the optical image, AFM image, height profile, and transfer characteristics of the device with 85-nm thickness, respectively. Figure S4. Hysteresis behavior of transfer characteristics of multi-layered MoTe2 transistor. Figure S5. (a)-(d) show the transfer characteristics of four multi-layered MoTe2 transistors, respectively. The black line represents the transfer characteristics of as-prepared multi-layered MoTe2 transistor in air, and the red line represents that measured from the annealed device. Figure S6. (a)-(c) show the transfer characteristics of three multi-layered MoTe2 transistors at different conditions, respectively. (DOCX 1011 kb) [file 11671_2018_2721_MOESM1_ESM.docx]

Additional file

Conversion of Multi-layered MoTe_2_ Transistor between P-type and N-type and Their Use in Inverter

*Junku Liu^1*^, Yangyang Wang^1^, Xiaoyang Xiao^2^, Kenan Zhang^3^, Nan Guo^1^, Yi Jia^1^, Shuyun Zhou^3^, Yang Wu^2^, Qunqing Li^2^, Lin Xiao^1*^*

*^1^Nanophotonics and Optoelectronics Research Center, Qian Xuesen Laboratory of Space Technology, China Academy of Space Technology, Beijing 100094, China*

*^2^State Key Laboratory of Low-Dimensional Quantum Physics, Department of Physics & Tsinghua-Foxconn Nanotechnology Research Center, Tsinghua University, Beijing 100084, China*

*^3^Department of Physics, Tsinghua University, Beijing 100084, China*

*Email addresses：*

*Junku liu：liujunku@qxslab.cn*

*Yangyang Wang: wangyangyang@qxslab.cn*

*Xiaoyang Xiao: xiao-xy13@mails.tsinghua.edu.cn*

*Kenan Zhang: zhangkenan1993@gmail.com*

*Nan Guo: guonan@qxslab.cn*

*Yi Jia: jiayi@qxslab.cn*

*Shuyun Zhou: syzhou@mail.tsinghua.edu.cn*

*Yang Wu: wuyang.thu@gmail.com*

*Qunqing Li: qunqli@tsinghua.edu.cn*

*Liu xiao: xiaolin@qxslab.cn*

*Corresponding Authors:*

*J.L. (liujunku@qxslab.cn) and L.X. (xiaolin@qxslab.cn)*


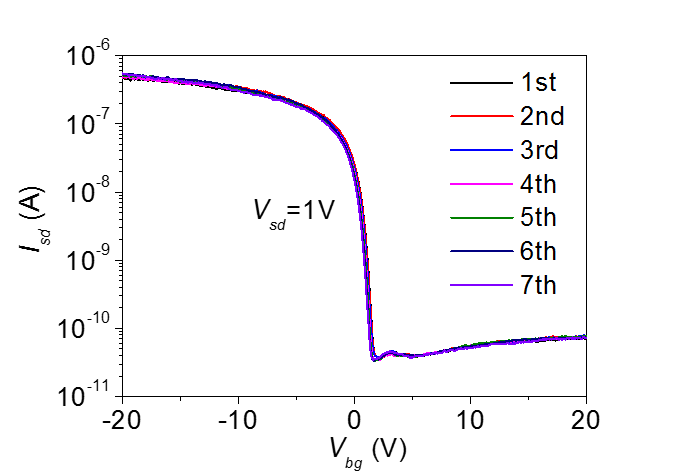


**Figure S1.** Transfer characteristics of replicate measurements


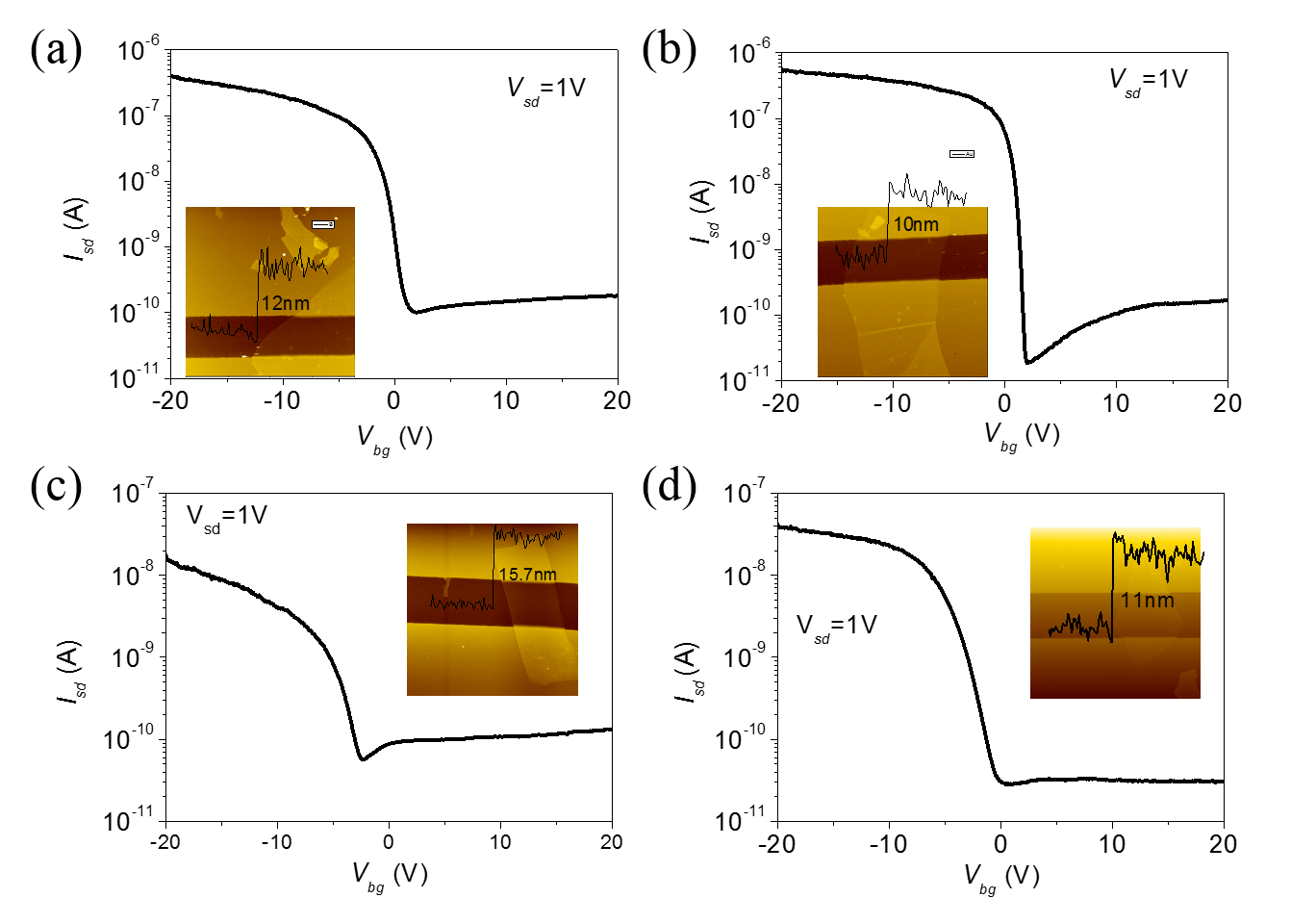


**Figure S2.** (a)-(d) show the transfer characteristics of other four multi-layered MoTe_2_ transistors in air, respectively. Inset image shows the AFM image and corresponding thickness.


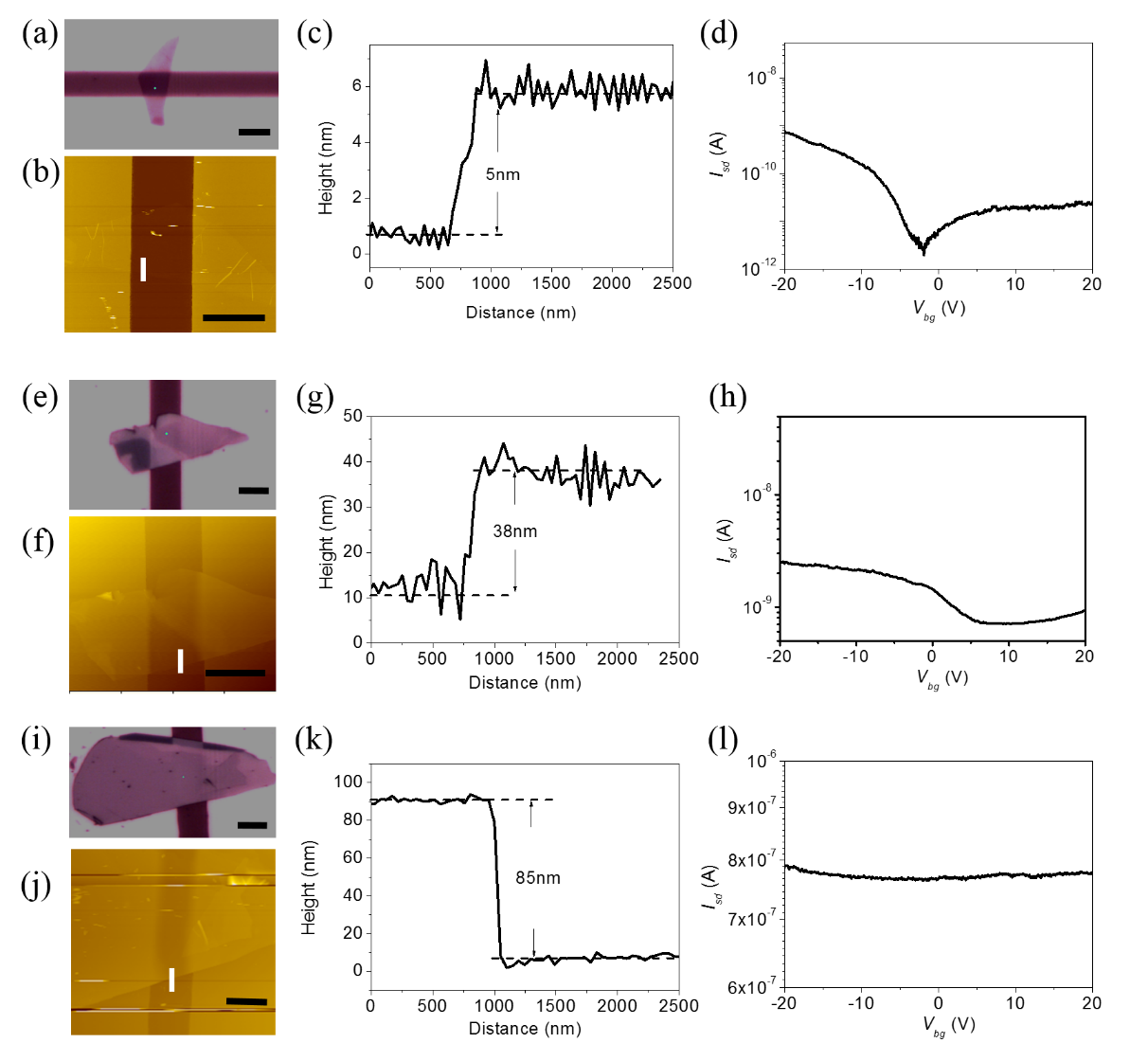


**Figure S3.** MoTe_2_ transistor with different thickness. (a)~(d) show the optical image, AFM image, height profile and transfer characteristics of the device with 5nm thickness, respectively. (e)~(h) show the optical image, AFM image, height profile and transfer characteristics of the device with 38nm thickness, respectively. (i)~(l) show the optical image, AFM image, height profile and transfer characteristics of the device with 85nm thickness, respectively.


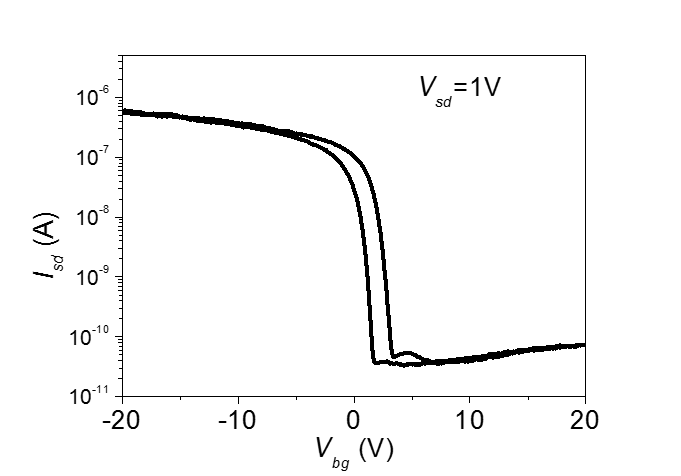


**Figure S4.** Hysteresis behavior of transfer characteristics of multi-layered MoTe_2_ transistor


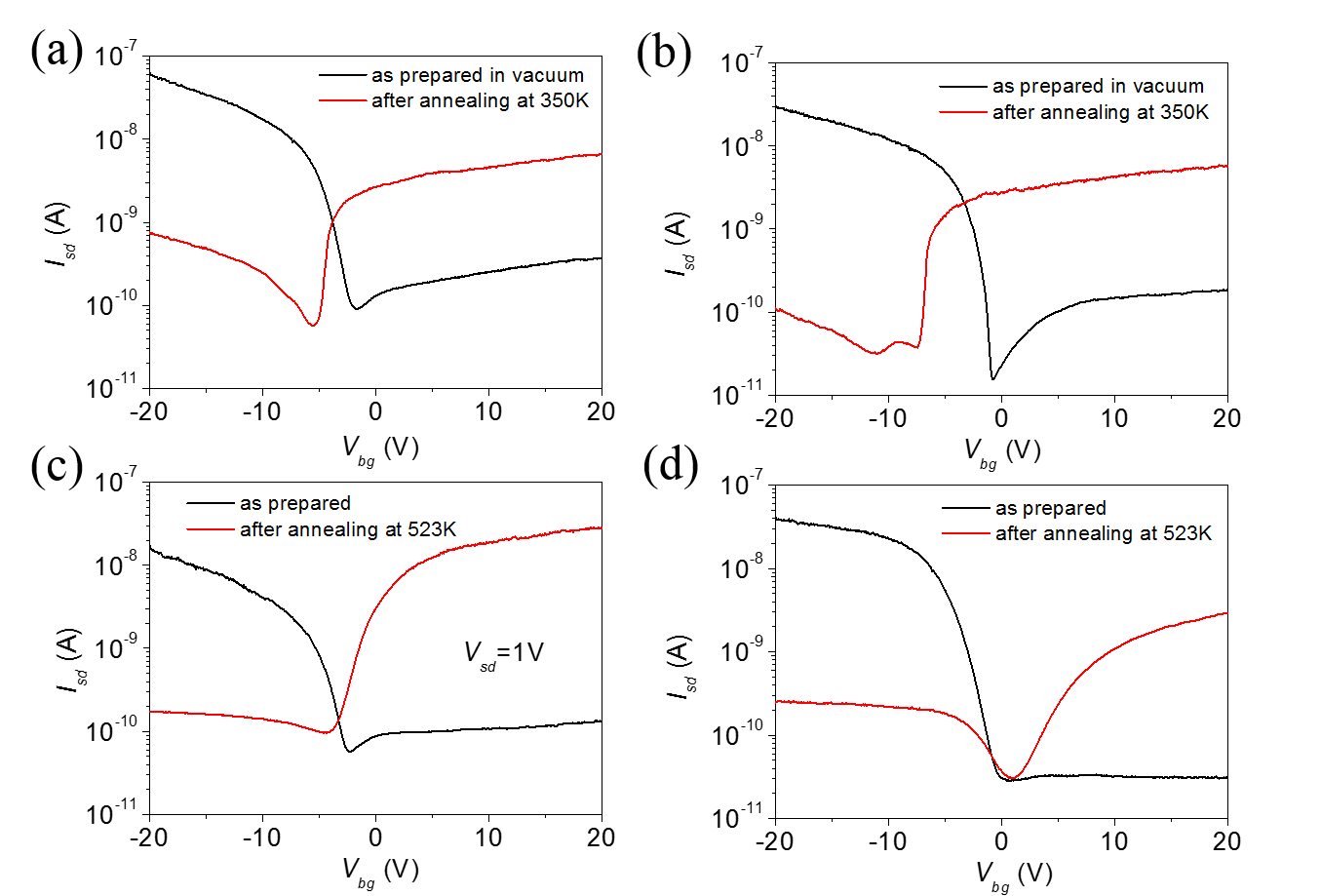


**Figure S5.** (a)-(d) show the transfer characteristics of four multi-layered MoTe_2_ transistors, respectively. Black line represents the transfer characteristics of as-prepared multi-layered MoTe_2_ transistor in air, and red line represents that measured from the annealed device.


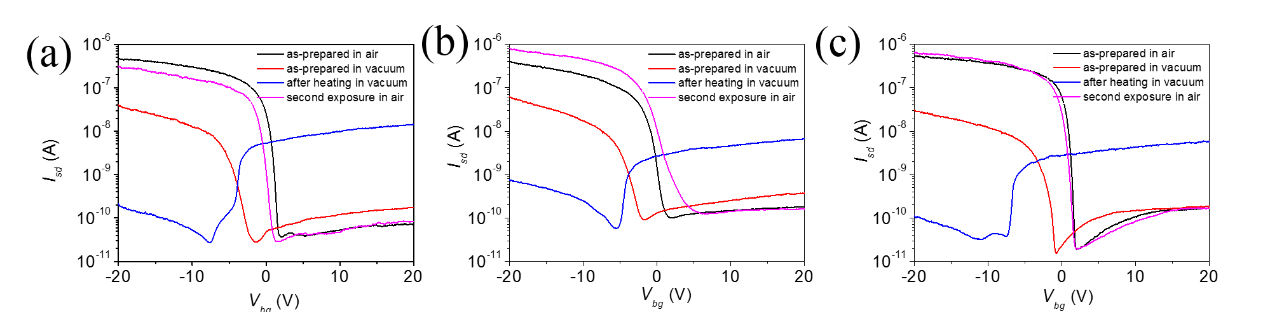


**Figure S6.** (a)-(c) show the transfer characteristics of three multi-layered MoTe_2_ transistors at different condition, respectively.
